# Temporal effect of docetaxel on bone quality in a rodent model of vertebral metastases

**DOI:** 10.1371/journal.pone.0320134

**Published:** 2025-04-17

**Authors:** Margarete K. Akens, Mohammedayaz Rangrez, Allison Tolgyesi, Thomas L. Willett, Cari M. Whyne

**Affiliations:** 1 Princess Margaret Cancer Centre, University Health Network, Toronto, ON, Canada; 2 Department of Medical Biophysics, University of Toronto, Toronto, ON, Canada; 3 Department of Surgery, University of Toronto, Toronto, ON, Canada; 4 Orthopaedic Biomechanics Laboratory, Sunnybrook Research Institute, Toronto, ON, Canada; 5 Institute of Biomedical Engineering, University of TorontoToronto, ON, Canada; 6 Department of Systems Design Engineering, University of Waterloo, Canada; Shanghai Jiaotong University: Shanghai Jiao Tong University, CHINA

## Abstract

This study investigates the effects of the anticancer drug docetaxel (DTX) and its timing of administration on tumor development and resultant bone quality in a rodent model, considering both healthy animals and those with osteolytic bone metastases secondary to intra-cardiac injection (d0) of HeLa cells. Healthy and tumor-bearing rats were treated with DTX on d7 or d14 and compared to the control (no treatment) and an additional cohort treated with Zoledronic acid (ZOL). Notably, DTX administration on d7 markedly curtailed tumor growth, as evidenced by bioluminescence and histological analysis, indicating its effectiveness in reducing bone metastases. Bone metastases were more established in animals treated with later DTX administration and ZOL, but still reduced compared to no treatment. When considering bone quality, we found that both the organic and mineral phases of bone are impacted by DTX treatment. Tumor-bearing animals exhibited decreased hydroxyproline/proline ratios reflecting change in collagen metabolism compared to healthy controls, but these decreases were only significant with no treatment or DTX administration on d14. This suggests a positive impact of early DTX treatment similar to ZOL on bone quality from an organic perspective. As well, increased CaMean and CaPeak reflecting the degree of calcification was found in healthy rats treated early with DTX, similar to that seen with ZOL compared to the tumor-bearing treated groups. Overall, early docetaxel administration reduced tumor formation and improved bone quality, suggesting its potential benefit in managing bone metastases.

## Introduction

Spinal metastases occur at high frequency in advanced prostate (90%), breast (75%), lung (45%), and renal (30%) cancer [1]. It can present as bone-destructive (osteolytic), bone-generating (osteoblastic), or a combination of both [2]. Osteolytic lesions are initiated by the interaction of the tumor cells with the bone matrix. It leads to activating the TGFß – PTHrP axis, stimulating osteoclast activity via RANKL[2]. Increased bone resorption leads to decreased bone mass and the weakening of trabecular structures, resulting in skeletal-related events in patients, such as fractures. Treatment outcomes and patient survival vary greatly depending on the progression of the metastases, with poor outcomes in advanced stages [3]. Advancing therapies that target spinal metastases requires understanding both effects on tumors and remaining bone quality. Systemic treatments include osteoclast inhibitors and chemotherapy to kill tumor cells [4].

Taxanes (paclitaxel and docetaxel) are a group of chemotherapeutic agents that inhibit microtubule functioning, leading to altered mitosis and cell death. Docetaxel, a second-generation taxane, is an antimitotic drug interfering with microtubulin assembly by stabilizing polymers against depolymeriztion, causing cell cycle arrest in the G2 phase and increasing apoptosis [5]. Docetaxel is widely used for a range of malignancies, including lung, breast, prostate cancers and bone metastases [4,6]. Taxanes generally activate mitogen-activated protein kinases (MAPKs) and cyclooxygenase-2 mRNA expression, increasing prostaglandin E2 (PGE2) production [7] and indirectly activating osteoclasts. However, taxanes inhibit osteoclast activation more than PGE2 indirect activation [8] affecting ruffled border formation and resorption vesicle transport, thus inhibiting bone resorption [9,10]

Bisphosphonates, drugs that impede bone resorption by osteoclasts, are classified into two main types: non-nitrogen-containing (simple) and nitrogen-containing bisphosphonates. The latter, including risedronate and zoledronic acid, contain nitrogen in its heterocyclic ring structure. These nitrogen-containing bisphosphonates inhibit farnesylpyrophosphate (FPP) synthase in the mevalonate pathway. Zoledronic acid is over 10,000 times more potent than simple bisphosphonate etidronate [11,12], making it a widely used treatment for spinal metastases [13].

While preclinical and clinical studies show docetaxel’s impact on cancer treatment and bone metastases, little attention has been given to its effects on bone quality. These effects should be considered in the context of bisphosphonates, which are widely used clinically. Bone-tumor interaction differs during early and advanced metastasis stages. This study will evaluate docetaxel’s impact on bone quality in early and advanced osteolytic bone metastases using an in vivo preclinical model and compare its effects on bone and tumor to bisphosphonate treatment.

## Materials and methods

### Animal model

In compliance with ethical standards, all animal studies were performed with institutional approval (University Health Network, Toronto, Canada; AUP 6044) and ARRIVE guidelines were followed. The animal protocol outlines humane endpoints, which include 20% weight loss and decreased overall well-being accessed by reduced grooming, lethargy, and hunched posture, among others. Five to six-week-old female Hsd:RHFoxn1^rnu^ rats (Envigo, Indianapolis, IN, USA) weighing 100±10gm were injected intra-cardiac with 1.8x10^6^ HeLa cells in 200µl medium under general inhalation anaesthesia (2% isoflurane in oxygen at 1.5l/min) on d0 [14,15]. The HeLa cells (originally mislabeled as MT-1 cells) were obtained from Dr. Engebraaten, Oslo, Norway [16]. Sixty-three rats were randomly allocated to tumor injection, control, and treatment groups (Table 1). Docetaxel Injection USP 10 mg/ml (Pfizer, Kirkland, QC, Canada) was diluted 10 times with 0.9%NaCl before the injection into the rat tail vein at 5mg/kg on d7 or d14. Zoledronic acid (Zometa^®^, Novartis, Montreal, QC, Canada) was injected subcutaneously in a single dose at 60µg/kg diluted in 0.9%NaCl on d7. Rats were anesthetized for all procedures (intracardiac injection, bioluminescence imaging, intravenous injections) and received analgesics after cell injection (Metacam^®^, Boehringer Ingelheim, Burlington, ON, Canada; 2mg/kg subcutaneously). They were monitored daily for food and water intake, overall well-being, and weighted weekly.

**Table 1:** Number of animals in treatment and control groups.

	Untreated	DTX d7	DTX d14	ZOL d7
Healthy rats (H)	8	4	8	8
Tumor cell injected rats (T)	10	9	9	7

DTX: docetaxel; d: day; ZOL: zoledronic acid

### Bioluminescence imaging

**Fig 1:**
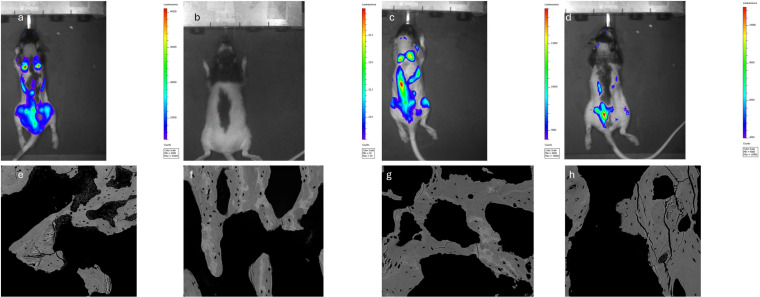
Bioluminescent images of the rats 21 days after tumor cell injection. (a: tumor, no treatment; **b:** **T** DTX d7; **c:** **T** DTX d14; **d:** **T** ZOL d7). The tumour burden in the **T** DTX d7 rat is so low that a signal could not be detected (note: in addition, the black skin underneath the black hair blocks photons from being detectable **(d)**). Backscatter electron microscopy images of the bone (e: tumor, no treatment; **f:** **T** DTX d7; **g:** **T** DTX d14; **h:** **T** ZOL d7). The **T** DTX d7 (f) bone shows the highest Ca distribution (CaWidth) variation reflected by the shades of gray. However, no statistically significant difference was found between the other groups.

Bioluminescent *in vivo* imaging was performed on d14 and d21 post-tumor cell injection to visualize the establishment of metastases and treatment effects (Fig 1(A-D)). Luciferin (XenoLight D-Luciferin, PerkinElmer, Woodbridge, ON, Canada) was dissolved in 0.9%NaCl (40mg/ml) and injected intraperitoneally at 90mg/kg. Thirteen minutes post-injection, the bioluminescent signal was acquired using a Xenogen IVIS 200 Imaging System (PerkinElmer) and analyzed with Living Image^®^ analysis software (PerkinElmer). A region of interest was drawn around the thoracolumbar spine, and results were expressed as photon/second/square cm per steradian (p/s/cm²/sr).

### Euthanasia and tissue harvest

Animals were sacrificed on d21 by CO_2_ asphyxiation. Thoracic vertebrae 11 (T11) and lumbar vertebrae (L5) were fixed in 10% buffered formalin for histological analyses and backscatter electron microscopy (BSE). Motion segments from T13 to L4 were frozen at -20°C for µCT imaging and microstructural analyses. L4 was used for high-performance liquid chromatography (HPLC).

### μCT imaging and microstructural analysis

As described in [17], excised T13–L4 segments were µCT scanned adjacent to hydroxyapatite (HA) calibration phantoms (µCT-100, Scanco Medical AG, Bruettisellen, Switzerland; Scan Parameters: 55kVp, 200μA, 11W, 34.9μm resolution). Trabecular bone within L2 vertebra was segmented using a validated semi-automated algorithm (AmiraDev 5.2.2; TGS, Berlin, Germany) [18]. The following structural parameters were analyzed from the vertebrae: Trabecular bone volume ratio (bone volume/total volume, BV/TV); %), Trabecular number (TbN; #/mm^2^), Trabecular spacing (TbS; µm), and Trabecular thickness (TbTh; µm), Trabecular volumetric bone mineral density (BMD; mg hydroxyapatite (HA)/cm^3^ calculated based on a HA phantom) and trabecular tissue mineral density (TMD; mg/cm^3^).

### Histological evaluation

Formalin fixed vertebrae were cut sagittal into two halves. One half was decalcified in 10% EDTA, and the other was embedded in resin for BSE analyses. Five-micron sagittal sections were stained with hematoxylin and eosin (H&E) to evaluate cell and tissue morphology. Human cytokeratin antibody staining the HeLa cell was used to evaluate tumor burden (Anti-wide spectrum Cytokeratin antibody (ab9377), Abcam, Waltham, MA) using HALO^®^ Image Analysis Platform (indica labs, Albuquerque, NM) and expressed in the % positively stained area of the analyzed section.

### High-performance liquid chromatography

HPLC measured mature lysyl oxidase-catalyzed collagen crosslinking in the L4 vertebrae tissue [15,19,20]. Briefly, all soft tissue was removed by blunt dissection and the L4 vertebrae were subjected to papain digestion to remove residual soft tissues. Samples were defatted and hydrolyzed with 11M HCl for 24hours at 110°C. The solution was diluted and added to a buffer with 10% acetonitrile, 1% HFBA, water, and an internal standard (pyridoxine). Pyridinoline, deoxy-pyridinoline, and pentosidine were quantified against standards of pentosidine (PolyPeptide Group, Strasbourg, France) and pyridinoline (Qiagen, Hilden, Germany). Agilent Zorbax Eclipse XDB-C18 Reversed-Phase C18 HPLC columns were used (150X4.6mm, 5mm particle size, 80Å pore size, end-capped; Agilent Technologies, Mississauga, Canada). The mobile phase included buffer A: 26%methanol + 0.1%HFBA, buffer B: 85%acetonitrile + 0.1%HFBA, and buffer C: 38%methanol + 0.08%HFBA, run at different elution times (A: 0–18min, 40–50min, B: 18–30min, C: 30–40min).

A separate HPLC method using a column of the same type measured collagen content via hydroxyproline quantification using hydroxyproline and amino acid standards (Sigma Aldrich, St. Louis, MI, USA). Samples were diluted with borate buffer with homoarginine (internal standard) and derivatized via the cycled addition of fluorenylmethyloxycarbonyl chloride (FMOC-Cl) + acetone, pentane, and extraction of the waste top layer. An elution profile measuring fluorescence versus elution time was obtained for each sample run. The areas under the peaks were measured and compared to a standard curve to calculate the concentration of each crosslink and the amino acids, hydroxyproline and proline. Crosslink concentration was normalized to collagen content and the extent of proline hydroxylation was determined by the hydroxyproline/proline ratio.

### Backscatter electron imaging

Quantitative BSE (qBSE) imaging was used to measure the distribution of calcium in bone tissue, indicative of mineral content. One-half of each L5 vertebrae (preserved in 10% buffered formalin solution) was subjected to sequential dehydration (acetone concentrations of 70%, 90%, 100%) and embedded in Spurr’s epoxy resin. The vertebrae were halved, and the surface microtomed and polished (paper grades of 400, 600 and 1200 grit size and diamond suspensions of 6 and 1µm). After carbon-coating the polished surface of each block, BSE microscopy was performed using a scanning electron microscope (SS BSE detector, FEI, Hillsboro, Oregon, US and FEI/Philips XL30, FEI, Hillsboro, Oregon, US). BSE grey levels were converted to calcium weight percentage [21,22] (Fig 1 (E-H).

### Statistical analysis

Data with normal distribution were analyzed using a one-way ANOVA with Šídák’s multiple comparisons (GraphPad Prism, GraphPad Software San Diego, CA, USA). Data from bioluminescence and histological analyses were unequally distributed and analyzed using Kruskal-Wallis with Dunn’s multiple comparison test. The level of significance was set at p<0.05.

## Results

### Clinical examination results

Tumor cell injection was generally well tolerated in 60 rats. One rat in each of the three tumor cell injected groups died before the experiment’s end (T-DTX d7: died 7d post tumor cell injection of unknown cause, T-DTX d14: died 13d post tumor cell injection of tumor development in the heart, and T-untreated: died after the injection with a wrongly diluted analgesic). Weight changes depended on tumor cell injection, tumor burden, and treatment time-point. The highest weight gain (36±5.2g) occurred in healthy untreated rats between d7 and d14 (2nd week), slowing to 9±4.4g between d14 and d21 (3rd week). Tumor-injected untreated rats gained 20±2.4g in the second week and lost 23±8.3g in the third week. Notably, docetaxel injection caused a reduced weight change within one week of injection (Table 2). Increasing tumor burden negatively influenced weight changes.

**Table 2:** Weight changes (Δ) in the rats two and three weeks after tumor cell injection and in the matching control rats. The rats were weighed on d7, d14 and d21.

Groups	Treatment Day	Weight Change (g) Week 2 (d14-d7)	Weight Change (g) Week 3 (d21-d14)
H untreated	–	36 ± 5.2	9 ± 4.4
T untreated	–	20 ± 2.4	-23 ± 8.3
H DTX d7	d7	3 ± 3.9	39 ± 6.2
T DTX d7	d7	4 ± 6.5	14 ± 2.8
H DTX d14	d14	15 ± 3.5	5 ± 2.4
T DTX d14	d14	14 ± 6.8	-3 ± 15.1
H ZOL d7	d7	13 ± 3.8	26 ± 12.0
T ZOL d7	d7	19 ± 2.8	-17 ± 18.0

H: healthy; T: tumor cell injected; d: day; DTX: docetaxel; ZOL: zoledronic acid

### Bioluminescent imaging

Tumor development was confirmed in 88.2% of the injected rats. A high photon count within the ROI, indicating tumor development in the spine, was observed in 9/10 rats in the T untreated group (median: 1.29E+07p/s/cm²/sr). A very low photon count (median: 3.70E+03p/s/cm²/sr) was measured in the T DTX d7 group in 8/9 rats, suggesting limited tumor involvement and lower tumor burden compared to the T untreated tumor group (p≤0.01). A medium-high photon count (median: 1.48E+06p/s/cm²/sr) in 7/8 rats was seen in the T DTX d14 group, suggesting less tumor suppression with later DTX administration. In the T ZOL d7 group, high photon counts (median: 5.08E+06p/s/cm²/sr) were seen in 6/7 rats.

### Histology

**Fig 2:**
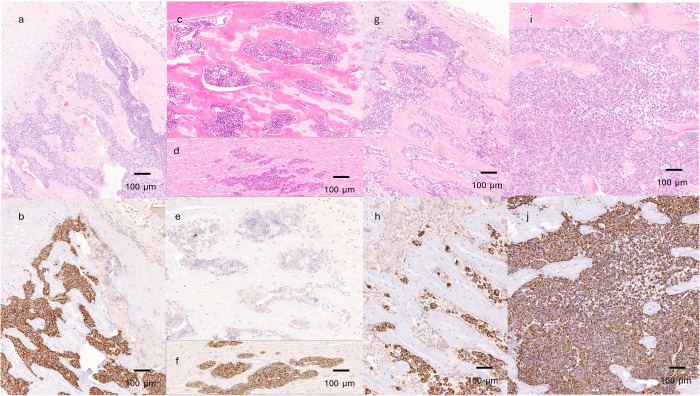
Sagittal images of vertebrae (a,b,c,e,g,h,i,j), and spinal cord (d,f) of the rats 21 days after tumor cell injection stained with hematoxylin and eosin (a;c;d;g;i) and immunohistochemical stain with an anti-wide spectrum cytokeratin antibody to identify the tumor cells (b,e,f,h,j). (a,b: tumor, no treatment; c,d,e,**f:** **T** DTX d7; g,**h:** **T** DTX d14; i,**j:** **T** ZOL d7).

In the histological evaluation of the T9 and L5 vertebrae, tumor cells occupied a median of 39.4% (range: 0–57.7%) of the analyzed area in the T untreated tumor-bearing control group. In contrast, only a few tumor cells were found in the T DTX d7 group (median: 0.2%, range 0.0–1.7%). Tumor cells occupied a median of 7.3% (range 0–41.8%) in the T DTX d14 group and 24% (range: 0–46%) in the T ZOL d7 group (Fig 2). These findings align with the bioluminescent imaging results. Tumor burden varied widely between vertebrae, and only two vertebrae per rat underwent histological analyses. Significant differences were found between the T untreated and T DTX d7 groups (p=0.0053) and the T untreated and T ZOL d7 group (p=0.0152).

### High-performance liquid chromatography

**Fig 3:**
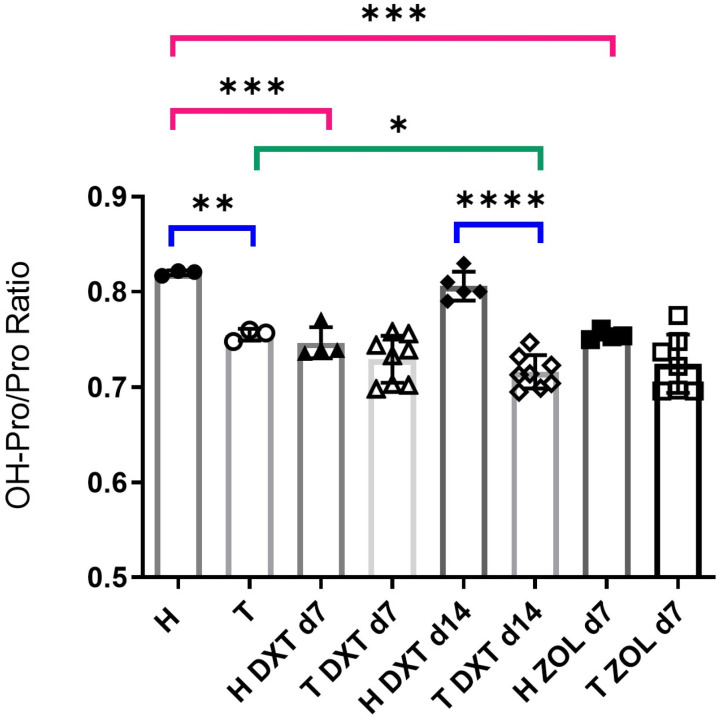
The hydroxyproline/proline ratio was significantly higher in healthy, untreated rats than in untreated, tumor-bearing rats. Significant differences between healthy and treated rats were also observed in the d14 DTX groups. In addition, healthy animals treated on d7 with either DTX or ZOL had a significantly lower ratio compared to healthy, untreated rats.

The hydroxyproline/proline ratio was significantly higher in H untreated rats vs. H DTX d7 (9%; p=0.0001) and H ZOL d7 (8%; p=0.0005) (Fig 3). This ratio was consistently higher in healthy rats than tumor-injected counterparts, with significant differences in the untreated and DTX d14 groups (7.9% and 11.2%, respectively). A significant decrease was also seen in tumor-bearing animals comparing T untreated to T DTX d14 (5.2%). Compared to the bone of healthy rats (H untreated), significant increases in hydroxyproline and proline concentrations were observed in the T untreated group indicating a change in collagen metabolism (note: increases in hydroxyproline (significant) and proline (not significant) with the presence of tumors were previously reported [15]). In contrast, hydroxyproline and proline concentrations were significantly lower in the bone of the T DTX d14 group compared to H DTX d14 (44% and 46%, respectively). A significant effect was seen only in Pentosidine (Pen) concentration when comparing the healthy and tumor groups receiving zoledronic acid treatment, where much higher pentosidine levels were observed in T ZOL d7 compared to H ZOL d7 (~400% increase).

### Backscatter electron imaging

CaMean measurements, representing the average degree of mineralization, increased in healthy animals with zoledronic acid delivery (H ZOL d7 vs. H untreated, 26.5%; p=0.021). A trend of increased mineralization was also observed with DTX on d7 (H DTX d7 vs. H untreated, 20%; p=0.084), but no effect was seen with DTX on d14 (H DTX d14, p=0.99). CaMean significantly decreased in T ZOL d7 vs. H ZOL d7 (21.2%; p=0.0036) and T DTX d7 vs. H DTX d7 (19.7%; p=0.019).CaPeak, the highest calcium content, increased in H ZOL d7 compared to H untreated rats by 31.8% (p=0.0057). D7 treatment groups showed lower CaPeak in tumor-bearing animals (H DTX d7 vs. T DTX d7, 17.8%; p=0.036 and H ZOL d7 vs. T ZOL d7, 23.9%; p=0.0057), but no differences were seen in untreated or d14 treated groups. CaWidth, the measurement of Ca content heterogeneity, showed no significant differences among groups. CaMean, CaPeak and CaWidth were not significantly affected by the treatments when comparing untreated tumor-bearing animals to tumor-bearing treated rats.

### Ex-vivo µCT imaging and analyses

In addition to microstructural differences reported in [17], this analysis focused on changes specific to docetaxel. We previously reported changes with ZOL treatment in tumor bearing vertebrae with significant increases in BV/TV, TbN and BMD and lower TbS compared to T untreated animals [17]. TMD was significantly higher in T DTX d7 vs. T untreated (12.2%). No other TMD differences were significant. BMD followed the TMD results, except T ZOL d7 had significantly higher BMD than T untreated (27.0%). BV/TV decreased with the establishment of osteolytic tumor. Higher BV/TV was observed in T DTX d7 (8.3%) vs. T untreated. TbN was higher in T DTX on d7 (9.7%) and d14 (11.1%) compared to T untreated. TbS decreased in T DTX d7 (15.1%) and d14 (13.2%) compared to T untreated. No significant TbS differences were found between healthy and tumor-injected animals treated with DTX at d7 or d14. TbTh decreased in T DTX d14 vs. H DTX d14 (5.0%) and untreated tumor group (6.1%). No differences were found comparing healthy DTX d7 or d14 groups with their tumor-injected counterparts. As reported [17], no morphological parameter differences among healthy animals were found across docetaxel treatment groups.

## Discussion

Docetaxel is often administered to patients with bone metastases. This study evaluated the effect of DTX on healthy rats and those with osteolytic metastases. The impact of DTX was further compared to the bisphosphonate Zoledronic acid, which is also widely used in patients with bone metastases. Docetaxel injection reduced weight gain in all treated rats—healthy and tumor-injected—for ~7 days post-injection. After this period, their weight changes re-aligned with their respective groups (healthy or tumor-bearing). This may be due to taxane-induced arthralgia and myalgia, typical side effects of docetaxel and paclitaxel in human patients [23]. Docetaxel stabilizes microtubules in cancer cells but also disrupts axonal transport in nerve cells, causing inflammation and oxidative stress [24]. It also increases COX2 expression, leading to higher prostaglandin E2 (PGE2) levels, which cause pain [7]. PGE2 interacts with osteoblasts and osteoclasts, either promoting bone formation via BMP2 activation or increasing resorption with increased osteoclast differentiation [7].

Tumor development, confirmed by bioluminescence, varied based on treatment type and timing post-inoculation. Photon counts, indicative of tumor size, were lowest in the T DTX d7 group, increased in the T DTX d14 and T ZOL d7 groups, and were highest in the untreated group. Histology confirmed these results. While these findings suggest early docetaxel treatment (d7) reduces bone metastases, a local tumor injection study (Luc CaP 23.1 injected into the tibia) found that higher and more frequent DTX doses showed no antitumor effect [25]. This difference may arise from immediate tumor establishment due to the local route of administration. Docetaxel given later (d14) had limited impact, indicating the importance of early treatment. Similarly, only a limited impact on tumor burden was seen with ZOL.

Microstructural parameters were influenced by tumor burden and treatment, as shown by Tolgyesi et al. [17]. In the T DTX d7 group, reduced tumor burden led to higher TMD, BMD, BV/TV, TbN, and TbTh (with lower TbS) in tumor-injected rats. Although the T DTX d14 group had more tumors than the T DTX d7 group, the tumor burden was lower than in untreated rats, resulting in higher TbN and lower TbS, but the trabeculae were thinner (lower TbTh). This indicates ongoing but slower degradation in the T DTX d14 group compared to untreated tumor-bearing animals.

Collagen changes in the bone due to treatment and tumor burden were assessed using HPLC [26]. The hydroxyproline/proline ratio was lower in all tumor-cell injected groups than in healthy controls, with significant decreases in untreated and DTX d14 cohorts. This contrasts with earlier findings of a slight (~6%) increase in this ratio in untreated samples with osteolytic tumors [15]. An increased rate of remodelling typically increases the hydroxyproline/proline ratio, only observed in the T untreated and T DTX d7 groups. The results indicate that DTX and ZOL treatments affect collagen metabolism. Like Olejnik et al., we found a decreased hydroxyproline/proline ratio in healthy rats treated with ZOL, possibly due to reduced proline hydroxylation [27]. ZOL’s effect on osteoblasts may involve post-translational modification of collagen, separate from its osteoclast inhibition. DTX influences the tumor microenvironment and upregulates the MMP1 gene, increasing collagenolytic MMPs [28,29]. In healthy animals, the hydroxyproline/proline ratio decreased with DTX on d7 but not on d14, suggesting a transient effect on MMP1 upregulation. Oxidative stress raises pentosidine (an advanced glycation end product (AGE)) levels, a negative bone quality marker released during high turnover. Pentosidine is a non-enzymatic cross-link that forms between adjacent collagen type I molecules [30]. ZOL inhibits pentosidine release by reducing osteoclast activity in healthy rats, but this effect is diminished by tumor presence due to reduced bone in ZOL-treated rats with osteolytic metastases [17].

The effects of treatment on hydroxyapatite (Ca5(PO4)3OH), the mineral phase of bone, were assessed using BSE. Increased calcification, indicated by higher CaMean and CaPeak, was observed in rats treated on d7, likely due to DTX and ZOL inhibiting osteoclasts, reducing bone resorption, and increasing calcium deposition [10]. Unlike Olejnik et al., we found significant increases in CaMean and CaPeak in healthy rats treated with ZOL, possibly due to differences in rat age, sex, and dosing regimens [27]. The H DTX d7 and H ZOL d7 groups showed higher CaMean and CaPeak than the H untreated group (significant for ZOL and trending for DTX at p=0.084), while H DTX d14 had similar values to H untreated, suggesting a time-dependent effect on bone remodeling. These differences were not seen in tumor cell-injected animals.

Our ZOL findings, consistent with previous reports, showed its effects on healthy and tumor-bearing bone. BSE analysis in H ZOL d7 rats revealed positive correlations between CaMax, CaMean, CaWidth, and TMD, BMD, TbS, TbTh, and negative correlations with TbN. These correlations were absent in tumor-bearing rats treated with ZOL. Adding to previously reported effects of ZOL on load to failure, microdamage, and tumor burden [17,31], this study showed ZOL’s impact on collagen metabolism and calcium deposition, more pronounced in healthy treated animals than in tumor-bearing ones. Unlike DTX, ZOL treatment did not cause weight changes related to neurological effects.

A limitation of this tumor cell injection model is the short timeframe between tumor establishment and the observation of treatment effects. Tumors take ~14 days to establish, and humane endpoints are reached by d21 post-injection. In addition, we only describe the effects of DTX on bone in rats with osteolytic metastases caused by HeLa cells, not in bone with osteoblastic or mixed metastases or those caused by other cancer cell lines. Initially mislabeled as a breast cancer cell line (MT-1), these HeLa cells reliably produce clinically relevant osteolytic bone lesions [16]. This model allowed us to examine the impact of early (d7) and later (d14) DTX treatment on tumour and osteolytic bone quality. Early DTX treatment reduced tumor development and improved bone properties, but its effectiveness diminished with a higher established tumor burden.

Multiple animal models exist to study vertebral metastases, including mice, rats and rabbits. Rat models provide a good balance between larger animals (rabbits) in which local treatments (such as radiation or thermal ablation) can be more easily applied and mouse models which are limited in the size of tissues available for analysis. The intracardiac injection model provided multiple vertebrae with metastases for analysis using different techniques (i.e., HPLC, BSE, histology). The rat vertebrae are sufficiently sized to enable sagittal cross-sectioning allowing histological analysis and BSE to be performed on adjacent sections. This allows for a reduction in the total number of animals required for study [32].

Previous research has considered the combination of high-dose ZOL and DTX in the context of a local mouse bone tumor model [25] showing it to be more effective in controlling tumor volume than ZOL or DTX alone. While we evaluated ZOL and DTX at clinically relevant doses in the current study, we did not consider combination treatments. While such combinations are used clinically, this is less common; ZOL is often used prophylactically or in metastatic disease to reduce osteoclast activity and bone loss, whereas DTX is used primarily to treat active disease. ZOL and DTX administration schedules often do not align clinically creating barriers with respect to scheduling and overburdening patients with clinical visits. Future studies may consider potential additive or synergistic effects of DTX and ZOL at clinically relevant doses and under conditions of longer-term administration with aligned dosing schedules.

DTX treatment affects the organic and mineral phases of bone, with the impact varying by treatment timing and tumor burden. Early DTX administration reduces tumor formation and maintains bone quality despite temporary weight loss. While bone quality was improved in animals treated with ZOL delivered at d7, tumor cells remained within the marrow space. Although not as beneficial to bone quality as ZOL, DTX none-the-less reduces osteoclastic activity. This is essential information for patients already at risk of fragility fractures. Later DTX administration provided modest improvements with respect to tumor burden (similar to ZOL) and ongoing but slower degradation of the bone compared to untreated tumor-bearing animals, thus motivating early clinical consideration of DTX administration for maintenance of bone quality as well as tumor management. Overall, this study shows the potential of early DTX treatment on bone quality in the management of patients with or at risk of bone metastases.

## Supporting information

S1 File Data files to manuscript 'Temporal Effect of Docetaxel on Bone Quality in a Rodent Model of Vertebral Metastases', https://doi.org/10.5683/SP3/MTHK1O, Borealis, V1.  (JPG)
