# Supplementary figures and images for: Temporal effect of docetaxel on bone quality in a rodent model of vertebral metastases
Source: PLoS One. 2025 Apr 17;20(4):e0320134. doi: 10.1371/journal.pone.0320134 (PMC12005523; doi:10.1371/journal.pone.0320134)

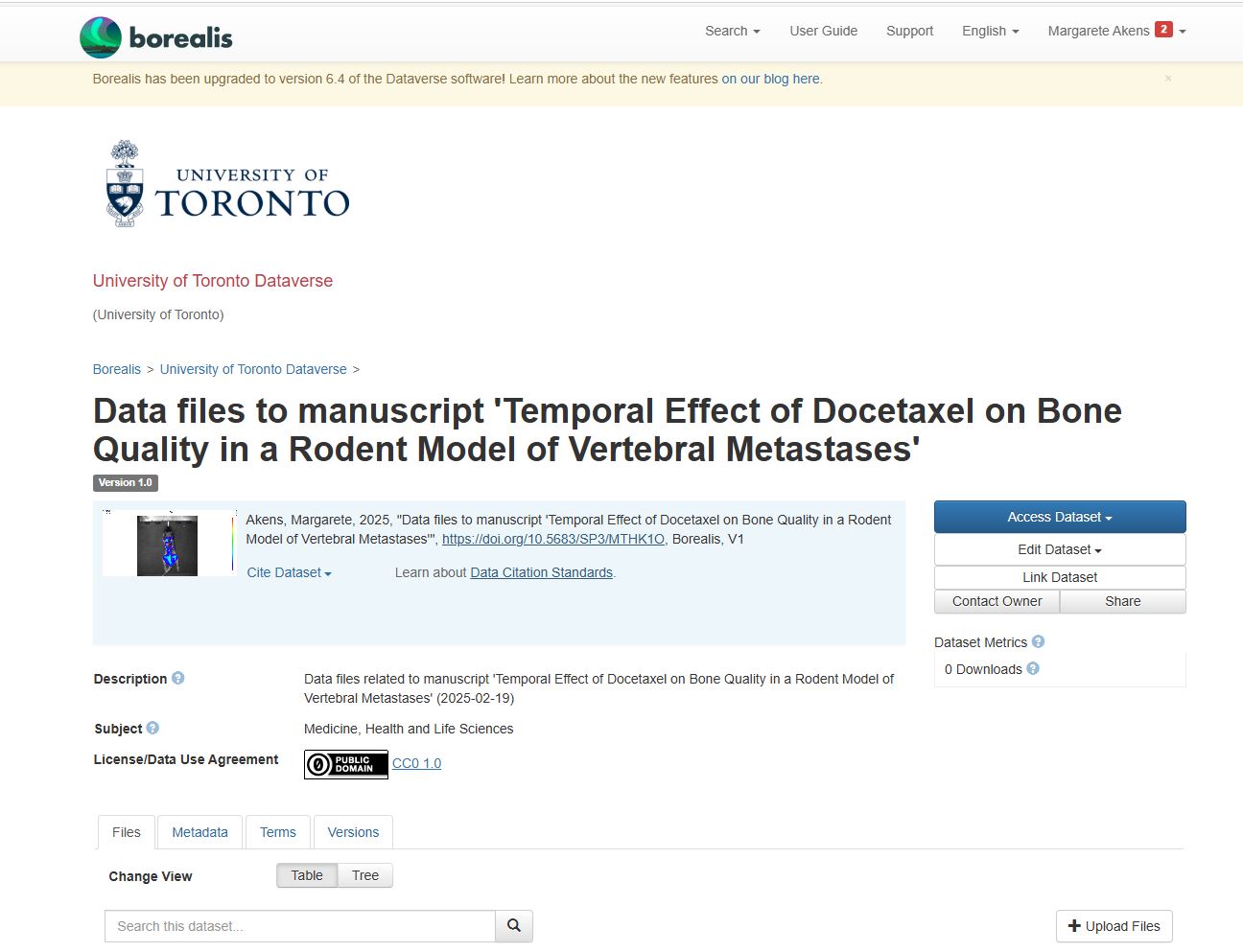

Supplement: S1 File — (JPG) [file pone.0320134.s001.JPG]
